# Circadian Phase Resetting via Single and Multiple Control Targets

**DOI:** 10.1371/journal.pcbi.1000104

**Published:** 2008-07-04

**Authors:** Neda Bagheri, Jörg Stelling, Francis J. Doyle

**Affiliations:** 1 Department of Electrical and Computer Engineering, University of California Santa Barbara, Santa Barbara, California, United States of America; 2 Institute of Computational Science and Swiss Institute of Bioinformatics, Zurich, Switzerland; 3 Department of Chemical Engineering, University of California Santa Barbara, Santa Barbara, California, United States of America; Indiana University, United States of America

## Abstract

Circadian entrainment is necessary for rhythmic physiological functions to be appropriately timed over the 24-hour day. Disruption of circadian rhythms has been associated with sleep and neuro-behavioral impairments as well as cancer. To date, light is widely accepted to be the most powerful circadian synchronizer, motivating its use as a key control input for phase resetting. Through sensitivity analysis, we identify additional control targets whose individual and simultaneous manipulation (via a model predictive control algorithm) out-perform the open-loop light-based phase recovery dynamics by nearly 3-fold. We further demonstrate the robustness of phase resetting by synchronizing short- and long-period mutant phenotypes to the 24-hour environment; the control algorithm is robust in the presence of model mismatch. These studies prove the efficacy and immediate application of model predictive control in experimental studies and medicine. In particular, maintaining proper circadian regulation may significantly decrease the chance of acquiring chronic illness.

## Author Summary

The robust timing, or phase, of the circadian clock is critical in directing and synchronizing molecular, cellular, and organismal behaviors. The clock's failure to maintain precision and adaption is associated with sleeping disorders, depression, and cancer. To better study and control the timing of circadian rhythms, we make use of systems theoretic tools such as sensitivity analysis and model predictive control (MPC). Sensitivity analysis is used to identify key driving mechanisms without having to fully understand or investigate the detailed mechanistic interconnections of the large complex circadian network. Contrary to intuition, sensitivity analysis of the circadian model highlights several *non*-photic control inputs (such as transcriptional regulation) that outperform light-based circadian phase resetting – light is known to accelerate protein degradation. Aside from targeting individual parameters as control inputs, our methods identify combinations of control targets that may further the efficiency of entrainment. We compare the phase resetting performance of our MPC algorithm among cases involving individual and multiple simultaneous control targets (in wild-type simulations). We then tailor the algorithm to correct continuously the phase mismatch that occurs in short and long period mutant phenotypes. Through use of the presented tools, our algorithm is robust in the presence of model mismatch and outperforms the natural *in silico* sun-cycle–based phase recovery strategy by nearly 3-fold.

## Introduction

Control theoretic tools have been used to model mRNA transcriptional/translational regulatory feedback mechanisms [1], to analyze nonlinear phenomena [2],[3], and to control complex biological behavior [4],[5]. In our research, we couple systems theoretic tools (such as sensitivity analysis) with model predictive control, to better address phase resetting properties of nonlinear biological oscillators. Our work aims to alleviate circadian-related disorders (such as jet lag and advanced/delayed sleep phase syndromes) by investigating the phase resetting properties of an example circadian mathematical model. More specifically, we manipulate multiple control inputs (or target parameters) to drive the dynamic behavior of the system.

Many researchers have shown that the systematic application of light pulses may reset the phase of circadian clocks. This light pulse (input) to induced phase-shift (output) mapping is most notably characterized by the phase response curve (PRC). Daan and Pittendrigh studied the PRC to establish a relationship among circadian behavior (nocturnal vs. diurnal activity), free-running period, and maximum phase advance/delay [6]. The free-running period of an organism reflects its circadian behavior without the influence of entrainment factors such as environmental light∶dark cycles. The free-running period of nocturnal animals, for instance, is often less than 24 hours such that dusk triggers a phase delay and the onset of activity. Conversely, diurnal animals often exhibit free-running periods greater than 24 hours such that dawn triggers a phase advance and the onset of activity [6]. Other researchers have made use of PRCs to establish light as a means to accelerate circadian entrainment [7], or as a means to start, stop, and reset the phase of simplified circadian models [8]–[11].

In a previous study, we develop a closed-loop nonlinear model predictive control (MPC) algorithm that minimizes the phase difference between a reference and a controlled system (each modeled as a single deterministic oscillator) through the systematic application of continuous light. Through use of MPC, circadian phase is recovered in almost half the time required by the natural open-loop sun cycles [4]. Next, we investigated how the MPC algorithm's tuning parameters might affect the model's phase resetting dynamics [12]. Here, we make use of sensitivity analysis to identify additional control inputs (or drug targets) that, when used by the MPC algorithm, outperform light-based circadian phase resetting. The target identification of single and multiple control inputs, coupled with the analysis of their respective performance, parallels efforts in the pharmaceutical industry to yield the greatest behavioral response with respect to the smallest system perturbation. In other words, our methodology may be used to identify optimal (and arguably non-intuitive) drug targets for therapy.

To establish an upper bound relating to the time required to recover phase differences, we begin by evaluating the open-loop control algorithm in the *Open-Loop Phase Recovery* section. The identification and manipulation of a set of single, dual, and triple control inputs are then used to minimize phase recovery dynamics of a wild-type circadian system (as described in the *Single*, *Dual*, and *Triple Target Phase Resetting* sections, respectively). This case is most similar to resetting a healthy organism's phase when subject to an environmental disturbance such as jet lag. In the *Short and Long Period Mutants* section, we further investigate how MPC may be used to alleviate chronic circadian disorders. More specifically, we apply the algorithm to circadian oscillator models that exhibit either short or long-period mutant phenotypes. Results suggest that organisms with such syndromes may track regular 24 hour rhythms through the systematic application of light. Our findings support this unique application of systematic drug target identification coupled with model predictive control for use in medicine and pharmacology (see the *Discussion* section). In the *Methods* section, we describe the employed model predictive control algorithm and the state-based sensitivity analysis used to identify single and multiple parametric control inputs.

## Results

A 10-state, 38-parameter *Drosophila melanogaster* (fruit fly) circadian model serves as the example system. This stable nonlinear limit cycle oscillator consists of two coupled negative feedback loops that characterize the transcriptional regulation of *period* and *timeless* mRNA and protein dynamics [13]. *per* and *tim* genes are transcribed in the nucleus, after which their mRNAs are transported into the cytosol where they serve as a template for protein synthesis. The doubly phosphorylated proteins form a heterodimer, PER-TIM, that enters the nucleus and inhibits gene expression, closing the feedback loop. Researchers find that environmental light increases the rate of TIM protein degradation: in this model, light targets the system by magnifying *ν* *_dT_*, the doubly phosphorylated TIM protein degradation rate [13].

**Figure 1:**
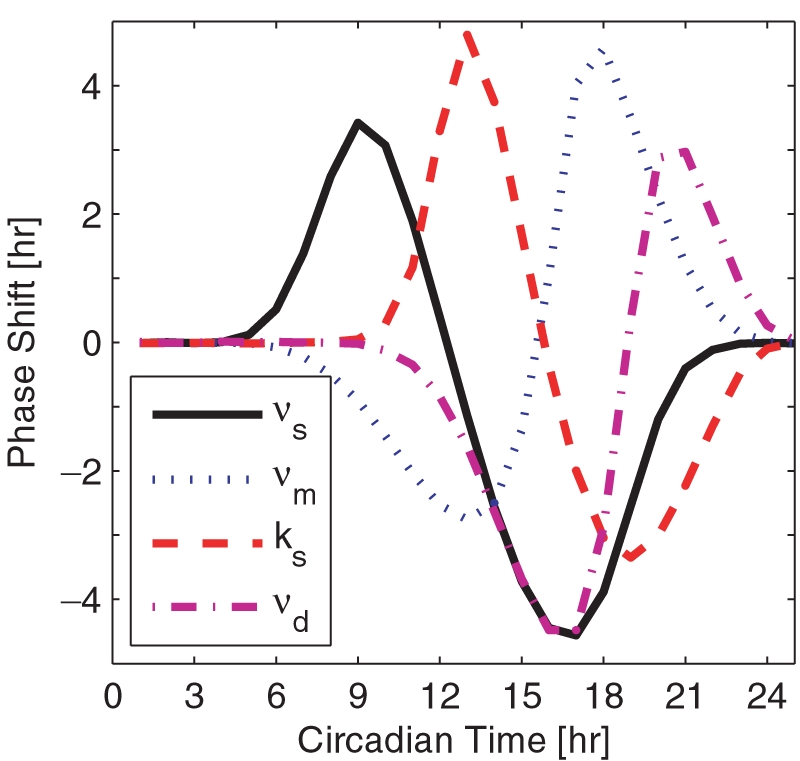
Circadian phase response behavior. Phase response curves traditionally characterize the light pulse to induced phase mapping of the input admitted to a free-running circadian oscillator. Here, phase response dynamics of the four system parameters exhibiting greatest state sensitivity is depicted: *ν_s_* (mRNA transcription), *ν_m_* (mRNA degradation), *k_s_* (protein translation), and *ν_d_* (protein degradation). The x-axis denotes the time at which the 2 hour pulse is given (where CT0 reflects dawn and CT12 dusk), and the y-axis describes the induced phase shift. A positive shift reflects a phase advance. Since light targets TIM specific protein degradation, *ν_dT_*, the light-based PRC of the *Drosophila* model is represented via the dash-dotted line.

The phase response of this model as a function of light is shown via the dash-dotted line in Figure 1. This curve maps the *circadian time* of the entraining stimulus (light pulses) against the resulting change in phase of an organism kept in a free-running environment. The circadian time index repeats every 24 hours with CT0 defining the commencement of dawn and CT12 that of dusk. It is important to note that the magnitude of light-induced phase changes (the quantitative dynamics of the PRC) may vary with respect to the intensity of light. While this model does not account for the complexity of the real network that, for instance, includes additional positive feedback loops [14],[15], it has been experimentally validated [15] and is widely employed as a reference model [3],[16].

### Open-Loop Phase Recovery

**Figure 2:**
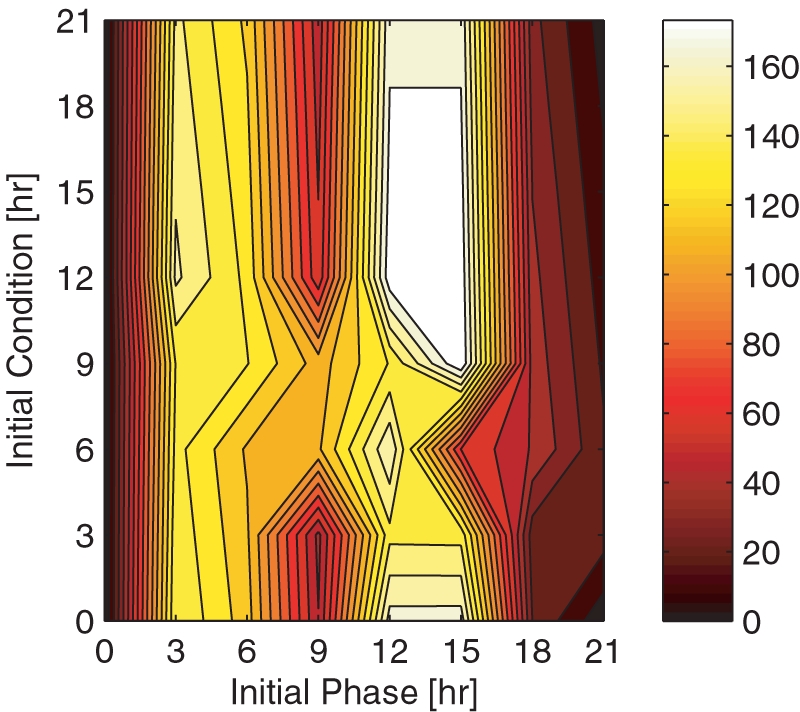
Natural phase entrainment. Open-loop phase resetting dynamics are plotted as a function of the initial phase difference (x-axis) and the initial condition (y-axis). The intensity of the color reflects the amount of time required to reset a given phase via the light∶dark cycles calibrated to the initial condition: the lighter the color, the longer the recovery time. The mapping of color intensity to phase recovery times (in hours) is shown in the vertical color bar.

Due to the inherent nonlinear phase response of circadian rhythms when subject to environmental/parametric perturbations, phase recovery dynamics are characterized as a function of the *initial condition* (IC, the circadian time at which control or entrainment begins), and *initial phase difference* (IP, the amount of circadian time to be recovered). To establish a phase resetting set point or upper bound (the maximum amount of time required to recover a given phase difference), we evaluate the open-loop control algorithm, where environmental light∶dark cycles serve as the only mechanism for phase re-entrainment. The phase recovery surface (Figure 2) displays the time required for the open-loop case to recover from any possible initial condition and initial phase difference. The asymmetry of the surface may be attributed to the nonlinear effects of light, as characterized by the PRC. The input (light) to output (induced phase shift) mapping of the PRC is seldom symmetric. In *Drosophila melanogaster*, a 15 minute pulse of light has shown to induce up to 3.6 hours of phase advance and 4.2 hours of phase delay [13]. Recent studies suggest that the change in phase is less sensitive to the duration of the light, and more sensitive to its time-profile [17]. Phase recovery times (for both open and closed-loop simulations) are evaluated with respect to initial conditions and phase differences discretized at 3 hour intervals. Thus, given the integers *i*,*j* ∈ [0,7], IC = 3*i* and IP = 3*j*.

The open-loop entrainment strategy requires at most 183 hours to reset the observed states of the controlled system (cumulative protein complex concentrations) to within 15% of the reference trajectories. Mandating the convergence of state trajectories is a tighter constraint than mandating only phase trajectories, since it incorporates amplitude characteristics. The algorithm, however, may be tuned to consider only strict phase measures. The maximum open-loop recovery time refers to a 9 hour initial phase difference whose control action begins at an initial condition of 15 hours. The initial condition, or start of entrainment, is described with respect to circadian time (CT). Interestingly, there is a stark difference between resetting a 3 to 6 hour initial phase difference versus an 18 to 21 hour initial phase difference (a −6 to −3 hour phase difference). In the former, phase recovers in over 100 hours; in the latter, phase recovers in fewer than 60 hours. Additionally, the open-loop algorithm recovers 9 hour phase differences in a fraction of the time required to correct for smaller phase difference. These properties may be attributed to the nature of the phase response curve and are discussed further in the *Discussion* section. Experimental studies in mammalian SCN cells support this asymmetry: Reddy *et al.* show that circadian clock resetting from a 6 hour phase advance (IP6) is accompanied by dissociation of cellular gene expression and may take up to 1 week to recover [18]. Conversely, resetting a 6 hour phase delay (IP18) is accompanied by coordinated gene expression and requires only 2 days of recovery [18]. Our simulations support these experimental conclusions as the cumulative protein concentrations in the former case diverge and require several days to converge to the nominal trajectory. In the latter, cumulative protein concentrations oscillate with smaller amplitude until they converge to the nominal trajectory within a couple days. An example of the corresponding simulations is presented in Figure S1.

### Closed-Loop Phase Recovery

The MPC algorithm (described in the *Model Predictive Control* section) minimizes the normalized difference between the cumulative protein complex concentration over a prediction horizon of 48 hours, by admitting control action during the first 8 hours of the simulated trajectory. This control action is multiplicative, allowing the algorithm to increase/decrease the nominal parameter by a factor of 2. The control profile defined within the move horizon is updated every 2 hours. Through use of MPC, the re-synchronization rate of the controlled system is increased nearly 3-fold through the control of light, or *ν* *_dT_*. Although light serves as a powerful control input, we show that the manipulation of parameters such as transcription and mRNA degradation rates (*ν* *_s_* and *ν* *_m_*, respectively) may provide more immediate phase resetting. Since we make use of the symmetric version of the mathematical model [13], we do not differentiate between *per* or *tim* specific functions. Instead, we assume that the isolated control of *ν* *_sP_* is equivalent to the isolated control of *ν* *_sT_*, for instance.

Parametric sensitivity analysis quantifies the relative change of system behavior with respect to an isolated parametric perturbation. A large sensitivity to a parameter, for instance, suggests that the system's performance is subject to greater change with small variations in the given parameter. We make use of the Fisher Information Matrix (FIM) to evaluate the effect of parametric perturbations on the circadian system's state trajectories [19]. Investigation of the diagonal values, off diagonal values, and singular value decomposition of the FIM points out the relative order, or rank, of parametric sensitivity measures. This relative ordering highlights sets of control inputs whose manipulation may further reduce phase recovery times. The three greatest diagonal values, for instance, identify the most prominent individual control targets (ranked from most to least sensitive);

- *ν* *_s_* (the mRNA transcription rate),
- *ν* *_m_* (the mRNA degradation rate),
- *k_s_* (the protein translation rate), and
- *ν* *_d_* (the doubly phosphorylated protein degradation rate).

Recall that *ν* *_dT_* is the target parameter of environmental light in *Drosophila*. Interestingly, the rate of mRNA transcription is the target of environmental light in *Mus* (via *per* genes) [20],[21] and *Neurospora* (via *frq* genes) [22]. Furthermore, in our previous studies of *Mus* and *Drosophila* circadian networks, mRNA transcription rates were among the most sensitive parameters with respect to both the state- and phase-based sensitivity analysis of two independent network representations [2].

The greatest off diagonal values identify the most prominent pairs of control targets (ranked accordingly);

- *ν* *_s_* and *ν* *_m_*, and
- *ν* *_s_* and *K_I_* (the threshold constants for repression).

Since the manipulation of more than 1 parameter voids the symmetry argument, we target *tim* specific parameters in the implementation of multiple control targets.

The greatest input directions of the singular value decomposition identify the most prominent set of three control targets (ranked accordingly);

- *ν* *_s_*, *ν* *_m_*, and *K_I_*, and
- *ν* *_m_*, *ν* *_d_*, and *k_2_* (the nucleus to cytoplasm rate of transport).

### Single Target Phase Resetting

**Figure 3:**
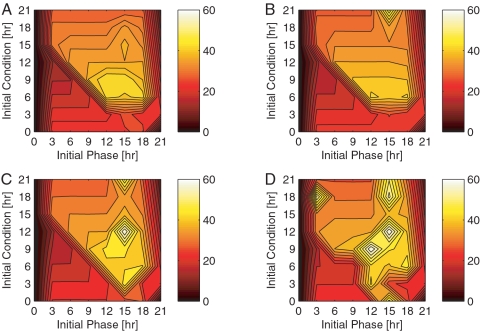
Single input control. Closed-loop phase resetting dynamics for single control targets (ordered according to their relative sensitivity) are described as a function of the initial phase difference (x-axis) and the initial condition (y-axis). The intensity of the color gradient reflects the amount of time required to recover from the given control conditions: the lighter the color, the longer the phase recovery. Each color bar is calibrated according to a minimum recovery time of 0 hours and maximum of 60 hours. (A) *ν* *_s_* Single Control Target (B) *ν* *_m_* Single Control Target (C) *k_s_* Single Control Target (D) *ν* *_d_* Single Control Target.

We investigate the phase recovery dynamics corresponding to four independent isolated control inputs with respect to the initial condition and initial phase difference (Figure 3). Results show that control targets identified via sensitivity analysis (Figure 3(A)–3(C)) serve as more effective re-entrainment factors than light (Figure 3(D)). More specifically, the maximum recovery time corresponding to a control input of *ν_s_* is 44 hours (at IC9 and IP12/IP15), *ν* *_m_* is 50 hours (at IC21 and IP15), *k_s_* is 59 hours (at IC12 and IP15), and *ν* *_d_* (the light target) is 60 hours (at IC12 and IP15, or IC9 and IP12). The control profiles and state response dynamics relating to the phase recovery of IC9 and IP12 are provided in Figure S2 and Figure S3.

There is a subtle similarity among the single-input phase recovery data; namely, the sudden drop in recovery time with respect to the initial condition for initial phase differences of 0 to 15 hours. We attribute this steep recovery gradient to the PRC as it depicts a greater region of phase delay than it does a phase advance. For this reason, it is more beneficial if the organism delays its phase to recover from a 12 hour initial phase difference. Furthermore, recall that a phase delay is incurred if the organism is to receive a photic input in the late evening hours. Hence, recovering from a phase difference via a set of delaying control inputs is most efficient if control action begins around the late subjective evening. Thus, if we observe phase resetting behavior corresponding to a small phase difference (such that the subjective day of the controlled system and reference are similar), we expect it to have the shortest recovery time near an initial condition of 12 hours, or dusk (Figure 3(D)). Interestingly, each of the control inputs exhibits this property. We attribute this similarity to the unique PRC of each control input (Figure 1).

#### Dual target phase resetting

**Figure 4:**
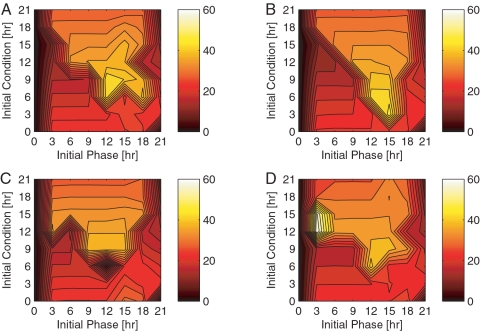
Multiple input control. Closed-loop phase resetting dynamics for dual ((A) and (B)), and triple ((C) and (D)) control targets are shown with respect to the initial phase difference (x-axis) and the initial condition (y-axis). The phase recovery time is denoted by the intensity of the color at each given data point: the lighter the color, the longer the recovery time. The mapping of color intensity to the recovery time (in hours) is reflected in the color bar. Each color bar is calibrated according to a minimum recovery time of 0 hours and maximum of 60 hours. (A) *ν* *_s_* and *ν* *_m_* Dual Control Targets (B) *ν* *_s_* and *K_I_* Dual Control Targets (C) *ν* *_s_*, *ν* *_m_*, and *K_I_* Triple Control Targets (D) *ν* *_m_*, *ν* *_d_*, and *k*_2_ Triple Control Targets.

Allowing the MPC algorithm to manipulate two variables simultaneously provides more immediate phase resetting since the controller has greater flexibility. For instance, the simultaneous use of *ν_s_* and *ν* *_m_* requires a maximum phase recovery of 43 hours to recover a 12 hour initial phase difference entrained from an initial condition of 6 or 9 hours (Figure 4(A)). Similarly, the simultaneous control of *ν* *_s_* and *K_I_* requires a maximum phase recovery of 46 hours to recover a 15 hour initial phase difference (Figure 4B).

Given that the common input is *ν* *_s_*, we expect the dual control input phase recovery dynamics to be just as good (if not better) than the results generated from the single *ν* *_s_* input algorithm. Although the dual control input strategy provides similar maximum phase recovery times, the greatest recovery time “plateau” is smaller. Therefore, the dual *ν* *_s_* and *ν_m_* input strategy is more effective at recovering an initial phase differences of 15 hours from IC6, while the *ν* *_s_* and *K_I_* pair is more effective at recovering a 12 hour initial phase difference from IC9 (compare Figure 3(A) to Figure 4(A)–4(B)). We would even argue that the recovery times associated with the dual input strategy may lessen if the genetic algorithm based optimizer were run over a greater number of generations. We limit the number of generations in the genetic algorithm – 15 for the single input, 75 for the dual input, and 250 for the triple input – to reflect the limited resources and time constraints evident in real world applications.

#### Triple target phase resetting

Just as the dual input case, we expect the triple input strategy to recover phase just as effectively as the single input strategy. The *ν_s_*, *ν* *_m_*, and *K_I_* input strategy requires at most 39 hours to recover an initial phase difference of 12 hours at IC9 (Figure 4(C)). Interestingly, the maximum recovery time corresponding to the use of *ν* *_m_*, *ν* *_d_*, and *k_2_* as simultaneous control inputs is 59 hours to recover a 3 hour phase difference at IC12 (Figure 4(D)). We attribute this abnormally high recovery time to the possibility of numerical errors associated with the optimization algorithm since *ν* *_m_* requires no more than 30 hours to recover from a 3 hour phase difference, while *ν* *_d_* requires no more than 54 hours. If we omit this data point as an outlier, this triple input strategy requires 42 hours to recover a 12 hour phase difference beginning at IC6. Assuming abundant computational resources and time, the triple input strategy may further outperform the dual input strategy since the MPC algorithm acquires greater flexibility (a greater number of control options) with each additional target. Each of these control inputs produces a unique PRC that allows the algorithm to further manipulate the set of targets such that the combination may yield a phase delay or advance at any time of the circadian day. In the case of a single light (or *ν* *_d_* target) input, for instance, the algorithm must wait for the subjective morning to force a phase advance, or the subjective night to force a delay. The advantage gained through additional control targets, however, is not clear. Given the finite horizon over which the algorithm optimizes phase synchrony, in addition to the nonlinear response of the model, we can not expect a monotonic improvement of phase recovery dynamics with an increase in the number of manipulated variables. For instance, the algorithm may choose a sequence of multiple inputs that yields lower cost in the short term (as compared to a single input) with a greater cost in the long term, leading to a point of no return. This scenario may also attribute to the 59 hour recovery observed in Figure 4(D).

### Short and Long Period Mutants

**Figure 5:**
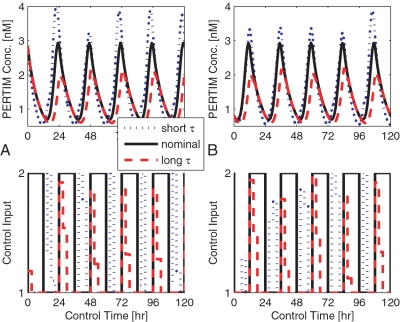
Model mis-match. Continuous phase resetting for the short-period mutant phenotype (dotted lines) and long-period mutant phenotype (dashed lines) are depicted with respect to an initial condition of 0 and 12 hours. Upper subplots describe the observed state trajectory (cumulative PER-TIM protein complex concentrations) as a function of controlled light pulses, shown in the lower subplots. The nominal response (denoted by solid lines) is entrained via regular 24 hour light∶dark cycles. As expected, short-period mutants reset via daily light pulses that occur during the subjective night, forcing daily phase delays. Long period mutants reset via daily light pulses that occur during the subjective morning, forcing phase advances. (A) Mutant Response at IC0 (B) Mutant Response at IC12.

Mutant phenotypes of the circadian oscillator represent cases in which nominal light∶dark cycles are unable to maintain synchrony. For this reason, the MPC tuning parameters must be re-evaluated according to this phase resetting problem. In wild-type, for instance, we can afford to be more aggressive with control penalties since nominal light∶dark cycles (or, no control) will eventually entrain the system. In mutants, the weights used to penalize the state error and control inputs prove to be more influential since nominal light∶dark cycles will not entrain the system. Therefore, we set both the move and prediction horizon to 24 hours and reduce the penalty of state error and control to ones. To counter the computational expense incurred with a longer move horizon, we set the time step to 4 hours. Through MPC, we identify a more suitable light∶dark cycle that synchronizes organisms exhibiting abnormally short and long free-running periods (22 and 27 hours, respectively, as shown in Figure 5). Determining the complete range of entrainment (which is likely wider than the 22 to 27 hour period) is non-trivial. In a previous study, we found that (i) the predicted range of entrainment may be very sensitive to the employed performance metric, and (ii) the control/light input strength may also play a dominant role in defining the bounds of this range [23].

Given that the PRC characterizing the behavior of *Drosophila melanogaster* consists of phase delays during the late subjective evening, we expect short-period mutant phenotypes to require bright light after subjective dusk. Similarly, we expect long-period mutant phenotypes to require bright light in the early subjective morning to advance the cycle. Our results confirm this hypothesis. In Figure 2, we demonstrate how bright light, admitted during the environmental night, resets the phase of short-period mutants such that it matches that of its environment. Given that the controlled system is 2 hours short, the occurrence of light during the night overlaps with the advance region of the system's PRC. Similarly, the onset of bright light at dawn overlaps with the delay region of long-period mutant PRCs (Figure 2). Our ability to maintain appropriate phase relationships between mutant phenotypes (models characterized by non-nominal parameters) and the environment (the nominal case) further proves the robustness of the algorithm despite model mismatch.

## Discussion

### Circadian Phase Response

As implied by the PRC (Figure 1), a 3 hour phase difference may be recovered immediately through admission of light at CT15. Hence, for open-loop control action to be most effective, environmental daylight should occur during the controlled system's subjective night (at CT15). In cases with small initial phase difference (such that the subject's internal time is nearly equal to environmental time), however, daylight begins entrainment once the subjective day is around CT12, by inducing small phase delays. This delay reduces the overlap between environmental daylight and the subjective night since re-entrainment of the initial phase difference began before subjective night. The opposite occurs with small *negative* phase differences, where an 18 hour (or −6 hour) phase difference may be recovered via a light pulse admitted at CT21. In this case, environmental daylight affects the controlled system at the start of day while it has not yet begun entrainment, maximizing the phase advancing effect of light. For this reason, open-loop entrainment via phase advances requires less recovery time despite the fact that a single pulse of light may induce a greater phase delay than advance.

More generally, we find that any given initial phase difference is more readily recovered if open-loop entrainment begins between CT0 and CT9; the rate of re-entrainment depends on the initial condition. To correct initial phase differences of 0 to 9 hours (by inducing a phase delay), daylight is most effective at the end of the day, suggesting greater performance if the algorithm were to begin control action around CT6. To correct initial phase differences of 0 to −6 hours (by inducing phase advances), daylight is most effective at the start of the day, suggesting greater performance if the algorithm were to begin around CT0. In the former case, daylight overlaps with the delay region of the subject's PRC, while in the latter it overlaps with the advance region. Resetting an initial condition of 12 to 15 hours, however, presents an interesting control dilemma as environmental daylight may induce both a phase delay and phase advance. For this reason, the open-loop control algorithm requires several days to correct for such phase differences. If light were accessible to entrain the system continuously throughout the day and night (in other words, if we were to *close the loop*), phase recovery dynamics would be less extreme since phase resetting would rely less on the initial condition.

Additional phase resetting properties may be inferred through investigation of the simulated PRCs. For instance, in the single input case, *ν* *_s_* and *k_s_* exhibit similar recovery dynamics with the exception that *ν* *_s_* is more effective at resetting initial phase differences of 15 to 21 hours. This quality may be associated with the fact that manipulating *k_s_* exhibits a strikingly similar phase response as *ν* *_s_* where their input to output mapping is shifted by about 5 hours (Figure 1). This similarity may be attributed to the fact *k_s_* and *ν* *_s_* are directly involved with the irreversible production, and transcriptional/translational regulation, of clock-specific genes/proteins. Additionally, the “active” region of the *ν* *_s_* and *ν_m_* PRCs are wider than those of *k_s_* and *ν* *_d_* (or, their dead zones are shorter than those of *k_s_* and *ν* *_d_*), suggesting that their perturbation-induced phase shifts are accessible throughout a greater portion of the circadian day.

### Minimizing Control Action

**Figure 6:**
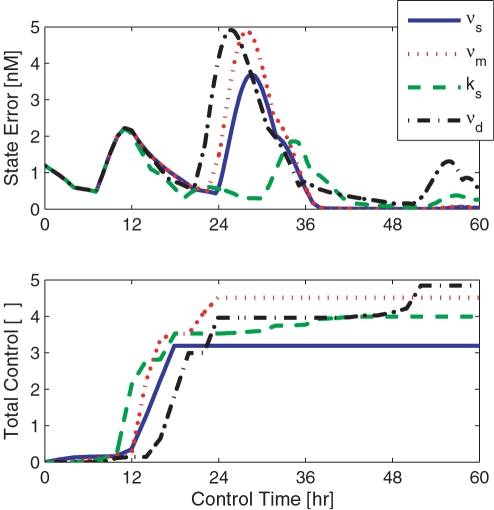
The cost of control. The convergence of state dynamics (from an initial phase difference of 15 hours at IC12) is plotted (top) as a function time. The corresponding cumulative control input (determined by integrating the value of each multiplicative control target over time) is described in the lower subplot. The light-induced target, *ν* *_d_*, shown via the dash-dotted line, requires the greatest amount of multiplicative control while exhibiting the greatest amount of state error. Conversely, the *k_s_* target corresponds to the least amount of state error and requires less admitted control.

Of the single control input results, the manipulation of *ν* *_s_*, identified as the most sensitive parameter, provides the shortest phase recovery times. Despite these results, *ν* *_d_* or light-based control is most efficient. In Figure 6, we relate the cumulative control input (a unitless measure that integrates the multiplicative control target action) to the convergence of phase via the PER-TIM complex state error. The data shown reflects the recovery of an initial phase difference of 15 hours from IC12. Analyzing this relationship may provide a basis from which the pharmaceutical industry might select one drug over another. If two different drug targets demonstrate similar response, the one that requires the least number of doses should be admitted, minimizing cost and the potential for drug related side-effects. Moreover, if the symptoms of illness are more severe than the potential for side effect, the drug that minimizes the state error may be preferred over others. The assessment of system convergence and the corresponding admitted control is key to the identification and application of control targets.

### Circadian Alignment and Illness

In our modern “24/7” work world, social and commercial pressures often oppose our natural circadian timekeeping, causing a source of circadian stress that may lead to chronic illnesses such as cardiovascular disease and cancer [24]. Numerous studies seem to show the effect of circadian rhythms on processes such as cell proliferation and apoptosis that eventually lead to proper growth control [25]–[27]. For instance, components of the cell cycle that dictate the G1-S and G2-M transition phase have been associated with circadian transcriptional regulation [28],[29]. Also in certain conditions, cancer can be a direct consequence of the absence of the circadian regulation [25],[26],[30]. A review of circadian related clinical disorders describes how mutations in some clock genes are associated with alcoholism, sleeping disorders, hypertension, and morbidity [24],[31]. Most commonly, poor circadian regulation leads to advanced sleep phase syndrome, delayed sleep phase syndrome, non-24-hour sleep-wake syndrome, and irregular sleep-wake pattern [32]. In each of these cases, poor circadian phase resetting may be achieved through the systematic admission of controlled light pulses.

Assuming we have access to drugs that specifically target circadian genes, we can identify the targets whose manipulation yields the most effective and immediate response through investigation of each control's phase dynamics (as shown in Figure 1). Or, it is possible to minimize the use of control and choose targets that require the least number of doses. We may also tailor the MPC algorithm to correct phase more readily through simultaneous manipulation of multiple control targets. Even further, we may reduce the computational expense by enumerating the control solutions over a grid in the solution space (light magnitude as a function of time), and choosing the optimal control sequence via an exhaustive search. The algorithm approaches a globally optimal solution as the total possible quantization steps of the control input increases. We tested the efficacy of the algorithm with respect to a quantization of 2, 4, 8, and 16 steps [12]. Results suggest that the shorter recovery time associated with the finer-grid enumeration may not outweigh the increase in computation time. Therefore, we may dramatically reduce computational expense by investigating control solutions for as few as 2 possible control values.

Our methods show great promise for use in the pharmaceutical industry as our theoretical phase entrainment of mutant phenotypes demonstrates the robustness of the algorithm in the presence of model mismatch. This robustness alleviates concerns in the pharmaceutical industry to tailor mathematical representations of bio-chemical pathways to individual people.

### Mammalian Studies

The study of controlled light pulses as a means of correcting phase is a common area of interest. Studies have shown that humans are much more sensitive to light than initially suspected since room light can significantly reset the phase of the human circadian clock [33],[34]. Furthermore, the admission of morning light has been considered as an antidepressant by realigning the internal clock with the environment [35].

Additional studies suggest that the human circadian clock mechanism functions similarly to those of other mammals [34]. This similarity may be attributed to shape/amplitude characteristics of their respective phase response curves. Humans show phase-delay shifts of up to 3.6 hours and phase-advance shifts of up to 2.01 hours (with respect to a 6.7 hour pulse of bright light) [36], which is both quantitatively and qualitatively similar to other mammalian species. This parallel motivates the experimental application of controlled light pulses for phase resetting in mammals. We have taken this first step by assessing the efficacy and computational utility of model predictive control as applied to a detailed 71-state *Mus musculus* circadian model [37]. Furthermore, melatonin has proven to be a key circadian phase resetting agent for totally blind people who cannot synchronize to environmental day∶night cycles (or do so at an abnormal time) [35]. Therefore, melatonin may be used individually (in cases to treat the totally blind), or in combination with light to provide more effective phase resetting.

Therapies designed to alleviate circadian load would have an important impact on morbidity and mortality across the developed world. Aside from correcting mutant phenotypes, phase resetting would increase performance in many healthy, or wild-type, cases such as frequent flyers avoiding jet-lag or astronauts maintaining a rigorous schedule during space exploration [17]. The real-time application of the proposed algorithm, however, may be a major issue; in practice, it will not be feasible to collect the corresponding protein concentration data at the molecular level. However, behavioral and/or physiological parameters that are controlled by (and correlated with) the circadian clock's dynamics are easily accessible. Such data may include actograms such as wheel running data for rodents [6]. Hence, a missing link in the current work concerns the development of corresponding (non-linear) state estimators for reconstructing the molecular dynamics. Given the discrete nature of MPC (sampling every 4 hours), the proposed strategy is feasible in practice since sampling rates of such physiological circadian markers may be much higher.

## Methods

A 10-state *Drosophila melanogaster* circadian limit cycle oscillator serves as the model system. This model consists of two coupled auto-regulatory transcription/translation negative feedback loops that characterize *period* and *timeless* gene and protein dynamics [13]. As demonstrated in previous work, the MPC algorithm may be applied to any stable limit cycle oscillator, including a more complex *Mus musculus* model [4]. Thus, we describe the example system as a general set of nonlinear ordinary differential equations with time *t*, *n*-length state vector **x**(*t*), environmental light input *l*(*t*), additional control inputs **u**(*t*), and system dynamics **f**(**x**(*t*), *l*(*t*), **u**(*t*)):
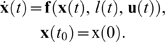
Given that both environmental light and additional control variables may be modeled as multiplicative inputs, the nominal wild-type (sun-cycle entrained) case requires *u*(*t*) = 1, while *l*(*t*) oscillates as a square wave with a frequency of 24 hours, between values 1 and 2. For consistency, the natural sun-cycle environment (or reference) is characterized by the nominal *Drosophila melanogaster* model and denoted by **r**(*t*). This reference is pre-entrained to normal 24 hour light∶dark cycles and is not subject to additional control inputs.

### Model Predictive Control

Model predictive control [38] is used to increase the re-synchronization or entrainment rate of circadian oscillators through the systematic application of specified control inputs. The algorithm follows a sample and hold strategy, updating the prediction and control input every *t_s_* = 2 hours, where the discrete time index 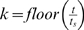, such that a function *g*(*kt_s_*) = *g*[*k*]. For simplicity, we refer to *k* as being equivalent to 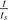 and ignore its rounding component. The manipulated control variable, *u*[*k*], optimizes an open-loop performance objective on a time interval extending from the current time 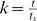 to the current time plus a prediction horizon of *P* = 48 hours, where 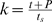. This horizon allows the algorithm to take control action at the current time in response to a forecasted error. The move horizon, *M* = 12 hours, limits the number of control inputs within the prediction horizon such that *u*[*k*] spans a time interval 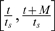. Beyond 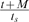 hours of simulation, the predictive model defaults to *u*[*k*] = 1. Future behaviors for a variety of control inputs are computed according to the mathematical model of the system [13].

The efficacy of the algorithm was evaluated with respect to a sample and hold time interval of 1, 2, and 3 hours (reflecting a move horizon of 3, 6, and 9 hours, respectively). Although shorter light pulses offer a more dynamic manipulated variable profile, it shortens the move horizon and may reduce the utility of model predictive control. Conversely, a longer pulse may reduce the possible control profiles since extended exposure to light leads to arrhythmic behavior [39]. Thus, we set the sampling rate to 2 hours.

The fitness function penalizes the normalized predicted state error between the reference and controlled trajectories, **ē**[*k*], and the net control, **ū**[*k*], over the prediction horizon. The system output used to evaluate circadian performance (or, phase entrainment) is the trajectory defined by the total *period* and *timeless* protein complex concentrations. This state error, **e**[*k*], is normalized with respect to the nominal amplitude of oscillation while the time dependent control input, **u**[*k*], is normalized with respect to the nominal set of values, 1:
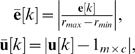
where the state dynamics **r**[*k*] characterize the nominal reference. Note that the vector **e**[*k*] is 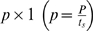, while the matrix **ū**[*k*] is *m*×*c* (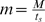 and *c* denotes the number of control inputs).

To avoid penalizing transient effects, the state error is weighted uniformly over the move horizon (reflected in the first *m* diagonal values of the *p*×*p* matrix **Q**), and with increasing weight of slope 2 over the prediction horizon (reflected in the *p*−*m* to *p* diagonal values of **Q**). The cost of applying a light input is weighted uniformly with a magnitude of 100 as reflected in the diagonal values of the *m*×*m* matrix **R**. We can afford to be conservative with the cost of control in the wild-type case, since we can ensure that the lack of control (the open-loop algorithm) will eventually entrain the system. The values contained in **R** will be re-evaluated when the algorithm is designed to entrain mutant phenotype models. The performance of an *m*-length control input is measured by
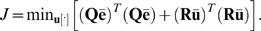
Only the first move of the lowest cost control sequence evaluated at time *k*, 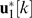, is implemented. Therefore, the sequence of actually implemented control moves may differ significantly from the sequence of control moves calculated at a particular time step. This discrepancy disappears as the prediction and move horizons near infinity. Feedback is incorporated by using the next measurement to update the optimization problem. Once the controlled state trajectories converge to within 15% of the reference state trajectories, the system is considered to have recovered its phase in *T_r_* = min*_k_*[|*e*[*k*]|_∞_≤0.15] hours. At this point, the algorithm defaults to no control since nominal light∶dark cycles will keep the system synchronized to the new environment. Optimization of the phase synchronizing control sequences is completed through use of a genetic algorithm [40]–[42].

### Sensitivity Analysis

Parametric sensitivity analysis quantifies the relative change of system behavior with respect to an isolated parametric perturbation. Parametric *state* sensitivity analysis assigns a value to each system parameter that defines how its perturbation affects state dynamics: 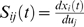. This tool is often used to identify the robustness and fragility tradeoffs of regulatory structures [3], and may be tailored to evaluate specific output performance such as period, amplitude, or phase characteristics [2].

Assuming the model has *n* states and *ρ* parameters, the FIM is a *ρ*×*ρ* matrix describing how any two parametric perturbations might affect state dynamics. More notably, the diagonal values of the FIM describe how any single parameter may affect state dynamics. As a result, we sort the values of the FIM from greatest to least magnitude and choose the top three individual parameters (reflected by the sorted diagonal values) and top three pairs of parameters (reflected by the sorted off-diagonals) whose perturbations yield the greatest change in output.

We further analyze the FIM via the singular value decomposition [43]. Assuming FIM = *F*, it may be decomposed as *F* = *UΣV^T^*, where Σ is an *n* by *p* diagonal matrix of non-negative singular values, *σ*, *n* is the number of states, and *p* is the total number of system parameters. Matrices *U* and *V* contain the eigenvectors of *FF^T^* and *F^T^F*, respectively. *U*, Σ, and *V* are ordered according to the magnitude of the singular values. Thus, the first column vector of *U* (and *V*) represents the output (and input) direction with largest amplification. The next most important direction is associated with the second column vector, and so forth. We determine the top three parameters associated with the three greatest input directions in *ν*_1_ and *ν*_2_ as ideal inputs for studying the multiple control input strategy.

## Supporting Information

Figure S1 Open loop phase resetting response at IC9. Phase resetting dynamics for an initial phase difference of 18 hours (or −6 hours) is shown in the blue dotted trajectory; those pertaining to IP6 are reflected in the red dashed line. The nominal protein concentration dynamics are depicted in the solid black line, while environmental sun cycles are shown in the black dotted square wave. The magnitude of the square wave oscillates between 1 and 2 and does not correspond to the y-axis of the figure.
(0.02 MB EPS)

Figure S2 Single control input to output response for IC9. Phase resetting dynamics (upper subplot) are depicted as a function of the individual control input profiles (lower subplot). Nominal, or pre-entrained, circadian dynamics are shown in solid black. The blue dotted lines reflect phase resetting with respect to *ν*s, while the red dashed lines reflect those of *ν*m. Although phase resetting, or state convergence, among the four different control variable occurs at similar hours, both the state dynamics and control profiles for each variable are significantly different.
(0.04 MB EPS)

Figure S3 Single control input to output response for IP12. Phase resetting dynamics (upper subplot) are depicted as a function of the individual control input profiles (lower subplot). Nominal, or pre-entrained, circadian dynamics are shown in solid black. The blue dotted lines reflect phase resetting with respect to ks, while the red dashed lines reflect those of *ν*d. Although phase resetting, or state convergence, among the four different control variable occurs at similar hours, both the state dynamics and control profiles for each variable are significantly different.
(0.03 MB EPS)
